# A dose-controlled system for air-liquid interface cell exposure and application to zinc oxide nanoparticles

**DOI:** 10.1186/1743-8977-6-32

**Published:** 2009-12-16

**Authors:** Anke Gabriele Lenz, Erwin Karg, Bernd Lentner, Vlad Dittrich, Christina Brandenberger, Barbara Rothen-Rutishauser, Holger Schulz, George A Ferron, Otmar Schmid

**Affiliations:** 1 Helmholtz Zentrum München, German Research Center for Environmental Health, Institute of Lung Biology and Disease, Ingolstaedter Landstrasse 1, D-85758 Neuherberg, Germany; 2 University of Bern, Institute of Anatomy, Division of Histology, Baltzerstrasse 2, CH-3000 Bern 9, Switzerland

## Abstract

**Background:**

Engineered nanoparticles are becoming increasingly ubiquitous and their toxicological effects on human health, as well as on the ecosystem, have become a concern. Since initial contact with nanoparticles occurs at the epithelium in the lungs (or skin, or eyes), *in vitro *cell studies with nanoparticles require dose-controlled systems for delivery of nanoparticles to epithelial cells cultured at the air-liquid interface.

**Results:**

A novel air-liquid interface cell exposure system (ALICE) for nanoparticles in liquids is presented and validated. The ALICE generates a dense cloud of droplets with a vibrating membrane nebulizer and utilizes combined cloud settling and single particle sedimentation for fast (~10 min; entire exposure), repeatable (<12%), low-stress and efficient delivery of nanoparticles, or dissolved substances, to cells cultured at the air-liquid interface. Validation with various types of nanoparticles (Au, ZnO and carbon black nanoparticles) and solutes (such as NaCl) showed that the ALICE provided spatially uniform deposition (<1.6% variability) and had no adverse effect on the viability of a widely used alveolar human epithelial-like cell line (A549). The cell deposited dose can be controlled with a quartz crystal microbalance (QCM) over a dynamic range of at least 0.02-200 μg/cm^2^. The cell-specific deposition efficiency is currently limited to 0.072 (7.2% for two commercially available 6-er transwell plates), but a deposition efficiency of up to 0.57 (57%) is possible for better cell coverage of the exposure chamber.

Dose-response measurements with ZnO nanoparticles (0.3-8.5 μg/cm^2^) showed significant differences in mRNA expression of pro-inflammatory (IL-8) and oxidative stress (HO-1) markers when comparing submerged and air-liquid interface exposures. Both exposure methods showed no cellular response below 1 μg/cm^2 ^ZnO, which indicates that ZnO nanoparticles are not toxic at occupationally allowed exposure levels.

**Conclusion:**

The ALICE is a useful tool for dose-controlled nanoparticle (or solute) exposure of cells at the air-liquid interface. Significant differences between cellular response after ZnO nanoparticle exposure under submerged and air-liquid interface conditions suggest that pharmaceutical and toxicological studies with inhaled (nano-)particles should be performed under the more realistic air-liquid interface, rather than submerged cell conditions.

## Background

Humans and other organisms are constantly exposed to a diverse set of exogenous substances. Ambient and occupational exposure to gases and particles are recognized as severe health risks, mainly via the lungs (inhalation), but also potentially via the skin or even the eyes [1]. In addition, the increasingly wide-spread use of engineered nanoparticles (diameter <100 nm in at least one dimension; there are currently standardization efforts under way (e.g. ONR CEN ISO/TS 27687:2009-06-01) applying this definition to "nanoobjects"), for medical imaging, new drug delivery technologies and various industrial products (such as sun screen, paint and water-proof clothing), for example, has also raised concern about the ecotoxicological and health impact of these nanoparticles [2-4]. For these types of particles, controlled exposure occurs via the skin, gastrointestinal tract and lungs as a result of cosmetic and medical applications. Oral application is a common non-invasive method of drug delivery and inhalation therapy shows promise not only for treatment of respiratory diseases, but also for drug delivery to the systemic circulation [5,6]. With the rapid development of nanotechnology, the use of nanoparticles as drug carriers or diagnostic tools has moved within reach [7].

*In vitro *studies on explants, isolated human cells or cell lines offer a powerful tool for studying substance effects directly on human biology without using animal studies or human volunteers. Traditionally, these *in vitro *experiments have been performed with *ex vivo *studies of isolated cells from extracted organs or biopsies under submerged conditions, where the reactive agent to be investigated is added to the culture medium, which completely covers the cells [8,9]. For primary contact organs such as the lung, the skin, or the eye, this represents an unrealistic way of exposure, since the *in vivo *exposure occurs at the air-liquid interface and not under fully immersed (submerged) conditions. Furthermore, submerged exposures may lead to interactions between the cell culture medium and the nanoparticles and to agglomeration of nanoparticles in the medium, which could affect the particle-induced biological response. Another disadvantage of submerged cell exposure to nanoparticles is that the motion of nanoparticles in liquids is mainly driven by random motion (diffusion) and not by directed sedimentation onto the cells as for larger particles [10,11]. Consequently, under submerged conditions a substantial fraction of the nanoparticles will either remain in the liquid or be lost to the lateral walls of the cell culture vessel, which alters the dose of nanoparticles interacting with the cells [11,12]. Direct exposure of the cells at the air-liquid interface has the advantage of minimizing these adverse effects, enhancing the pharmacological and/or toxicological insight gained from these *in vitro *experiments.

Several *in vitro *systems for cell exposure at the air-liquid interface have been described in the literature, however most of them were designed for exposure to dry substances such as cigarette smoke, freshly generated soot particles or medical and occupational (nano-)powders [13-17]. For liquid substances, other exposure systems are required. One of the few approaches reported in the literature uses a jet nebulizer for droplet formation combined with an Andersen cascade impactor for inertial droplet deposition on the cells, which are seeded on the impactor stages [18]. This system was intended to study the characteristics of aerosol delivery, stability, delivery efficiency, and expression efficacy of gene products for optimized inhalation gene therapy. The RHINOCON system was designed to use commercially available pump-spray units to spray liquid pharmaceutical formulations directly onto human pulmonary cells, for efficacy and toxicity testing [19]. The spray is released into an air flow directed at the cells onto which the spray droplets are deposited due to impaction. Similarly, Blank and coworkers [20] used a spray technique to deposit 1 μm polystyrene particles onto a human epithelial-like cell line (A549). All of these systems use impaction as the droplet deposition mechanism, which is likely to induce cellular stress due to the high flow rates and high speed collisions of the particles with the cells, and none of these devices provides direct measurements of the cell deposited substance dose.

In this study, a new exposure system (ALICE) is presented and validated, for dose-controlled delivery of nanoparticles in liquids or solutions to cell systems cultured at the air-liquid interface. The uniformity, efficiency, repeatability and accuracy of the exposure method is determined with various solutions and nanoparticle suspensions and its applicability to toxicological and pharmacological studies is verified by examining the response of a widely used human epithelial-like cell line (A549) after exposure to dilute salt solutions and zinc oxide nanoparticles.

## Materials and methods

### The air-liquid interface cell exposure system (ALICE)

#### Principle of operation

The ALICE utilizes cloud settling, in combination with single particle sedimentation, as the droplet deposition mechanism. *Cloud settling *(sometimes also referred to as *bulk motion of aerosol*) occurs when the droplet concentration is sufficiently high (dense cloud) to provide a large enough flow resistance to cause the air to go around, rather than through, the cloud of droplets. In this case, the entire cloud moves as an entity, at a speed significantly higher than the speed of an individual particle in the air, since only the outer rim of the cloud experiences drag forces, while the interior droplets experience no drag.

**Figure 1:**
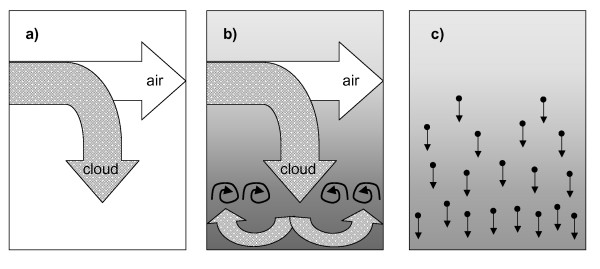
**Principle of operation of the air-liquid interface cell exposure system (ALICE)**. Three phases can be distinguished: During phase 1, a dense cloud embedded in an air flow is introduced into the empty chamber (panel a). During phase 2, the continuously supplied cloud forms a vortex near the bottom of the chamber and fills the chamber from bottom to top, while depleted air is extracted from the top part of the chamber (panel b). During phase 3, the flow and hence the influx of the cloud is stopped and the cloud -filled chamber is gradually depleted due to single particle settling (panel c).

The principle of the operation of the ALICE is schematically depicted in Figure 1. During phase 1 (Figure 1a), a dense cloud enters an exposure chamber entrained in an air flow near the top of the chamber. While the cloud settles rapidly to the bottom of the chamber (where the cells are located), the droplet-depleted air flow exits the chamber through the opposite side of the chamber. Near the bottom of the chamber the falling cloud gets diverted to all sides and forms an almost symmetric pattern of vortices (Figure 1b), which provides gentle, but sufficient, mixing to establish a spatially uniform cloud layer near the bottom of the chamber. With the continuous supply of cloud droplets, the chamber fills from the bottom up with the most dense cloud layer near the bottom (represented by the darker shading near the bottom of Figure 1b) and the lowest droplet concentration near the top (bright background). During the third phase (Figure 1c), the cloud (and air) flow is stopped and the droplets settle to the ground due to single particle settling. Of course particle settling is also active during phase 1 and 2, but its effect on cloud depletion is outweighed by the inflowing new cloud. Since the least dense part of the cloud is in the upper part of the chamber, extracting the air flow from this part of the chamber during phase 1 and 2 will not deplete the amount of droplets in the chamber very much.

The critical design aspects are: i) The cloud has to be dense enough for rapid "fall-out" so that most of the cloud remains in the chamber, ii) the air flow introducing the cloud into the chamber has to be chosen such that the falling cloud encounters the bottom of the chamber near its center (uniform distribution of the cloud) and the droplets have to be large enough for rapid single particle sedimentation to the ground. Since vibrating membrane nebulizers are characterized by high mass output and large particle diameter, this type of nebulizer is ideal for the ALICE.

The *cloud settling *speed can be calculated according to [21]

where *V*_*c*_, *m*_*c *_and *d*_*c *_are the speed, droplet mass concentration and diameter (characteristic dimension) of the cloud, respectively, *ρ*_*air *_is the density of air, *C*_*D *_is the drag coefficient (depends on particle Reynolds number) and *g *is the gravitational acceleration (9.81 m/s^2^). For an individual particle, the gravitational settling speed is given by [21]

where *V*_*p*_, *ρ*_*p*_, *d*_*p *_and *C*_*p *_are the speed, density, diameter and slip correction factor of the particle, respectively, and *μ *is the dynamic viscosity of air.

#### General setup

**Figure 2:**
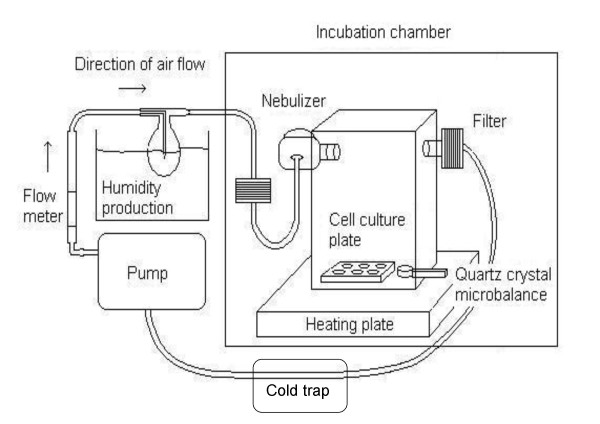
**Experimental setup of the air-liquid interface exposure system (ALICE)**.

The ALICE consists of four main components: 1) a *droplet generator *(nebulizer), which provides the dense cloud of droplets, 2) an *exposure chamber*, where the droplets deposit onto the cells located at the bottom of the chamber, 3) a *flow system with an incubation chamber*, which provides temperature and humidity conditions suitable for cell cultivation and 4) a *quartz crystal microbalance *(QCM) for real-time dose measurement (Figure 2). As seen in Figure 2, the droplets are generated by a nebulizer and transported by a humidified air flow into the exposure chamber.

#### Incubation chamber and air flow system

As seen in Figure 2, the ALICE system is operated with a closed loop flow system at a flow rate of 5 liter/min using an air pump and a flow meter. This flow rate was chosen as it transports the falling cloud to the center of the chamber, which is important for uniform spatial distribution of the aerosol in the chamber, as discussed below. The optimum cell culture conditions (T = 37°C, RH = 80-95%) are maintained by humidification of the air flow (sample air bubbles through 37°C water reservoir) prior to entering the nebulizer, and also by placing the nebulizer and the exposure chamber in an incubation chamber (polycarbonate, Makrolon™), which is thermally stabilized at 37°C using an RH/T-sensor (Model 177-H1, Testo, Germany) and a heating plate (PZ 230, Harry Gestigkeit GmbH, Germany) placed underneath the incubation chamber. The air flow exits the chamber through the opposite side of the entry port and is recirculated through a particle filter and a cold trap, where the former protects the pump and the latter avoids condensation of the water vapor in the "cold" parts of the tubing outside the incubation chamber.

#### Nebulizer (eFlow technology)

The liquid substance to be investigated is nebulized by a vibrating membrane generator (investigational eFlow, Pari Pharma GmbH, Germany), which was customized for the ALICE as described below [22,23]. This type of generator technology (TouchSpray™) utilizes a perforated, piezoelectrically-driven vibrating membrane to induce acoustic pressure waves, which periodically press small amounts of liquid through the tapered holes of a membrane. In the current study, the thin stainless steel membrane is perforated by about 3000 holes and vibrates at a frequency of 117 kHz. The nebulizer used in the current study has a reservoir chamber for spraying 0.5 to 5 mL of liquid, high liquid volume or mass output (up to 1.0 mL of liquid per min), a small residual amount of liquid in the reservoir (0.05-0.1 mL) and a narrow droplet size distribution (geometric standard deviation: 1.50-1.65) and it is characterized by a highly reproducible performance. The investigational eFlow switches off automatically after the liquid reservoir has been emptied. The nebulizer membrane was regularly cleaned by 5 min sonification in water. For zinc oxide (ZnO) and carbon black suspensions, the cleaning procedure was performed after every discharge in order to avoid partial clogging of the membrane pores which would result in reduced output efficiencies.

In contrast to the commercially available eFlow rapid, the applied investigational eFlow used i) a membrane with larger holes resulting in larger mass output and larger particles (here: mass median diameter MMD = 4.4-5.4 μm), which is important for rapid cloud settling and short droplet sedimentation time and ii) an aerosol chamber with an air inlet at the side and an aerosol outlet through the front (directly opposite of the membrane), which can be directly connected to the exposure chamber. With this setup, the distance between the vibrating membrane and the exposure chamber was 5 cm, the inner diameter of the connector to the chamber was 1.9 cm and the flow rate was 5 liter/min.

#### Exposure chamber

Exposure of the cells with the substance under investigation occurs in the exposure chamber, designed to hold up to two standard transwell plates (1 cm away from the walls of the chamber) containing cells cultured at the air-liquid interface. Immediately after exposure to the nebulized substance, the transwell plates can be easily removed (within a few seconds) from the chamber via a drawer for further analytical processing or post-incubation of the cells.

The exposure chamber is a 12 liter box (bottom plate: 20 × 20 cm^2^; height: 30 cm) made of polycarbonate (Makrolon™) with plates held in place by an aluminum frame. Makrolon is durable enough for repeated sterilization with alcohol and its transparent nature allows visual inspection of the motion of the cloud and the extent to which the chamber is filled with droplets (denser clouds appear more opaque). The highly concentrated cloud enters through the left side wall (in the center, 20 cm above the bottom) and gravitates swiftly to the ground (within ~1 s) due to *cloud settling*. The bulk motion of the cloud, which resembles "white smoke", can easily be observed with the naked eye. Using optical confirmation, the flow rate of 5 liter/min was chosen such that the falling cloud column reaches the bottom plate near its center in order to facilitate formation of an almost symmetric pattern of upwards vortices for uniform cloud mixing.

#### Quartz crystal microbalance (QCM)

Mass deposition onto the cells was measured with a quartz crystal microbalance (QCM 200/25, Stanford Research Systems, Sunnyvale, CA, USA) placed on the ground plate of the exposure chamber. The QCM is typically placed in one of the corners of the chamber, but the exact location is irrelevant, since the droplet deposition is spatially uniform, as is shown below. Mounting the QCM on a movable sledge allows for fast (within one second) and easy removal and insertion of the QCM, with minimum perturbation inside the exposure chamber.

The QCM determines the particle mass deposited onto a vibrating piezoelectric quartz crystal from the linear decrease in the resonance frequency of the crystal with increasing deposited mass. The QCM 200/25 uses a circular, AT-cut, α-quartz crystal with a resonance frequency of 5 MHz and operates at a sampling rate of 1 Hz. From the exposed crystal surface area, of 1.37 cm^2^, only the inner 0.4 cm^2 ^is active (experiences a displacement within the plane of the crystal). The change in mass per unit area (Δ*m*) is related to the observed change in oscillation frequency (Δ*f*) of the crystal by the Sauerbrey equation

where *C*_*f *_= 56.6 Hz cm^2^/μg (at room temperature) for a 5 MHz, AT-cut, α-quartz crystal. The Sauerbrey equation is valid only for uniform, rigid, thin films, where thin implies that Δ*f *< 10^5 ^Hz (2% of original resonance frequency) or Δ*m *< 1770 μg/cm^2 ^(equation 3). The sensitivity constant *C*_*f *_is a fundamental property of the crystal, so that the QCM does not require calibration [24].

For liquid films the Sauerbrey equation is not valid, since in this case the observed frequency change not only depends on the deposited mass of the film, but also on the viscosity and density of the liquid, as well as other factors such as the layer thickness, adsorption of material to the crystal and formation of sublayers within the film [24]. Hence, no simple linear dependence of frequency shift on deposited mass can be expected for liquid films, but an increase in Δ*f *is generally related to an increase in deposited mass, if the frequency shift remains below the asymptotic value for an infinitely thick layer of a given liquid. For water at 20°C, the asymptotic frequency shift is 715 Hz [25]. Hence, the QCM can be used as a real-time indicator for the deposition of droplets, but for accurate measurement of the cell deposited active substance (nanoparticles, solute), the liquid film has to be dried (here with dry air flow) and then interpreted using equation 3.

According to the manufacturer, surface-specific masses below 1 ng/cm^2 ^(Δf < 0.05 Hz) can be detected. Although this is close to the observed zero point stability of <0.1 Hz, a more conservative lower detection limit of 18 ng/cm^2 ^was adopted (Δ*f *= 1 Hz), since small temperature drifts can not be ruled out. The detection limit of18 ng/cm^2 ^corresponds to 25 ng of mass deposited on the exposed part of the QCM crystal (1.37 cm^2^). For ZnO nanoparticles used here, this corresponds to a uniform layer thickness of 0.03 nm or about 0.1 monolayers (density = 5.6 g/cm^3^).

### Substances used for ALICE characterization

Solutions and nanoparticle suspensions can be used in the ALICE. The characterization of the ALICE was performed with aqueous sodium chloride (NaCl), ammonium sulfate ((NH_4_)_2_SO_4_) and citrate solutions and with aqueous suspensions of gold (Au), ZnO and carbon black nanoparticles. The aqueous suspension of 15 nm Au nanoparticles, which was stabilized by 10 mM citrate (500 μg/mL), was purchased from British Biocell (EM.GC15, Batch 7894, British Biocell International, Plano GmbH, Wetzlar, Germany). The nominal particle concentration was 1.4 × 10^12 ^particles/mL (mass concentration: 40 μg/mL = 40 ppm with a gold density of 19 g/cm^3^). Additionally, a 10 fold enriched Au suspension was prepared by centrifugation of the suspension at 18,626 RCF (relative centrifugal force) for 20 min and by removing 90% of the (particle-free) supernatant. The ZnO and carbon black nanoparticle suspensions were prepared in our lab from commercially available powders (ZnO: AlfaAesar, Ward Hill, MA, USA Id# 43141; primary diameter: 24-71 nm (manufacturer information); BET surface area: 13 m^2^/g, agglomerated. Carbon Black: Printex 90, Degussa (now Evonik), Germany; primary diameter: 14 nm; agglomerated). The ZnO and carbon black suspensions were prepared, as well as vortexed and sonicated twice for 1 min intermittently immediately prior to spraying the suspension with the nebulizer.

### Characterization of uniformity and efficiency of droplet deposition in the ALICE

The uniformity of the droplet deposition in the exposure chamber was determined by placing 12 pieces of aluminum foil (3 × 3 cm) on the ground plate of the exposure chamber. Before and after exposure of the foils to the nebulized substances in the ALICE, their dry weight was determined by a gravimetric microbalance (Model r160p, Sartorius, Germany, accuracy ≤± 0.02 mg) and the deposited (dry) mass was determined from the change in foil mass. Adding all foil deposited salt masses, and scaling to the total area of the exposure chamber, yielded the total deposited salt (or nanoparticle) mass. The deposition efficiency was determined from the ratio of the total deposited (dry) mass and the mass filled into the nebulizer reservoir. The spatial uniformity of the deposition was determined from masses deposited onto the 12 foils. A qualitative representation of the spatial uniformity was obtained by transmission electron microscopy (TEM, CM12, FEI Co. Philips Electron Optics, Zürich, Switzerland) using a primary magnification of 3400× and 25,000× for Au and ZnO nanoparticles collected on TEM grids in the ALICE.

Time-resolved mass deposition was obtained for NaCl and (NH_4_)_2_SO_4 _solutions as well as for Au and ZnO suspensions by placing the QCM on the ground plate of the exposure chamber during an ALICE run.

For Au nanoparticles, the deposited mass was not only determined indirectly with the QCM (using the mass ratio of citrate and Au), but directly with gamma spectroscopy performed by the Helmholtz Zentrum Berlin in Berlin, Germany. The latter involved neutron activation of Au-197 into Au-198 (neutron flux was about 6 × 10^12 ^cm^-2^s^-1 ^for 1 hour) and subsequent determination of the Au mass on the aluminum foils from the intensity of the 412 keV gamma line of Au-198, relative to a known standard. As the obtained Au mass from QCM and gamma spectroscopy agreed within experimental uncertainties, both methods are considered equivalent.

### Cell exposure experiments

#### Preparation of salt solutions and nanoparticle suspensions

For the ALICE experiments, ZnO suspension of 0.3, 1.5 and 7.5 mg ZnO/mL sterile H_2_O (Braun, Melsungen) were prepared immediately prior to use from three stock suspensions of 1 mg/ml, 2 mg/ml and 10 mg/ml, respectively. The stock suspensions were produced, vortexed and sonicated twice for 1 min intermittently and then diluted with water to obtain 1 ml of ZnO suspension for nebulization. For control purposes, cells were also exposed to dilute (10 mM) aqueous citrate (stabilization agent in Au suspension) or NaCl solutions, which were also prepared immediately prior to nebulization.

For ZnO exposure under submerged conditions, the desired amount of ZnO was incorporated directly into the cell culture medium by adding the appropriate volume of a 1 mg ZnO/mL H_2_O stock suspension. Within 30 min ZnO agglomerates of about 900 nm (mobility diameter) had formed in the cell culture medium as determined by dynamic light scattering measurements (HPPS 5001, Malvern Instruments Ltd, Worcestershire, UK). As agglomerates of this size are known to efficiently deposit (near 100%) due to sedimentation [11], the cell deposited particle mass under submerged conditions was inferred, from the amount of ZnO mixed into the cell culture medium.

#### Cell handling for ALICE experiments

All exposure experiments were performed with a human epithelial-like cell line (A549) from a lung adenocarcinoma (obtained from ATTC, Manassas, VA, USA) representing the alveolar type II phenotype [26]. Cells were seeded into cell culture inserts (BD Falcon, transparent PET membrane, effective growth area 4.2 cm^2^, 1 μm pore size, 1.6 × 10^6 ^pores/cm^2^) with about 0.12 × 10^6 ^cells per cm^2 ^and cultivated under submerged conditions with DMEM/F12/L-Glut/15 mM HEPES buffer (Invitrogen, Germany) as culture medium, containing 100 Unit/mL penicillin, 100 μg/mL streptomycin and 10% fetal calf serum (FCS). The inserts were placed in BD Falcon™ 6-well tissue culture plates with 2 mL medium in the upper (insert) and 3 mL in the lower compartment. After 7 days of growth under submerged conditions at 37°, the cells had formed a confluent monolayer (0.3 × 10^6 ^per cm^2^). Subsequently, the cells were transferred to the air-liquid interface by removing the medium from the apical side of the cells and incubating them for another 18 h in the cell incubator. Following this procedure, it was shown that A549 cells closely resemble *in vivo *conditions by forming tight junctions and secreting a thin surfactant layer at the apical side of the cells [20].

Then the medium in the lower chamber was replaced with 3 mL serum-free culture medium and the cells were placed in the exposure chamber of the ALICE system for exposure to ZnO nanoparticles (or salt solutions) as described above. After 10 min in the ALICE, the cells were removed and incubated for 3 h in the cell incubator. Immediately after the post-incubation period, the cells were washed with PBS and directly lysed on the insert membrane by adding 350 μl of a cell lysis buffer, suitable for isolation of total RNA (Qiagen) (further details see RT-PCR section), or 2 mL WST-1 containing medium was added to the upper compartment (insert) to measure cell viability (further details are given below).

#### Cell handling under submerged exposure conditions

Adopting one of the most frequently used cell handling procedures for toxicological experiments [27,28], the A549 cells were seeded at 0.25 × 10^6^/cm^2 ^in 24-well plates (growth area 2 cm^2^) and incubated for 16 h in DMEM cell culture medium with FCS (see above) resulting in a cell density of approximately 0.4 × 10^6^/cm^2^. For ZnO exposure, the culture medium was replaced with 1 mL serum-free medium into which various amounts of ZnO particles (0.7, 2.5, 5 μg/cm^2^) were given by adding the appropriate volume of a 1 mg ZnO/mL H_2_O stock suspension. Subsequently, the A549 cells were incubated for 3 h. Biological parameters are reported relative to control conditions (incubated cell cultures without ZnO).

#### qRT-PCR analysis for analysis of IL-8 and HO-1 mRNA expression

Gene expression at the mRNA level of interleukin-8 (IL-8) and hemeoxygenase-1 (HO-1) was measured, 3 h after exposure, by quantitative reverse transcription polymerase chain reaction (qRT-PCR). The exposed cells were lysed and total RNA was purified using the Qiagen RNeasy Mini Kit (Qiagen GmbH, Hilden, Germany) according to the manufacturer's instructions. First-strand cDNAs were synthesized by reverse transcription from 0.5 μg total DNase I-treated RNA with a random nonamer primer (Metabion, Martinsried, Germany) and Superscript II reverse transcriptase (Invitrogen, Karlsruhe, Germany). For PCR amplification, the cDNA was mixed with the specific 5' and 3'primers and transcript levels were quantified using Absolute QPCR SYBR Green Mix plus ROX kit (ABgene, Hamburg, Germany) with the ABI Prism 7000 Sequence Detection System (Applied Biosystems, Foster City, CA, USA). The housekeeping gene GAPDH was used as internal reference to normalize the mRNA levels of the genes being studied, and IL-8 and HO-1 induction is reported after normalization to control conditions. The following primers were used: IL-8 5'primer: IL-8 5'primer: 5'-ATG ACT TCC AAG CTG GCC GTG GCT-3'; IL-8 3'primer: 5'-TCT CAG CCC TCT TCA AAA ACT TCT C-3'; HO-1 5'primer: 5'-AAG ATT GCC CAG AAA GCC CTG GAC-3'; HO-1 3'primer: 5'-AAC TGT CGC CAC CAG AAA GCT GAG-3'; GAPDH 5'primer: 5'-CCA TGA GAA GTA TGA CAA CAG CC-3'; GAPDH 3'primer: 5'-TGG CAG GTT TTT CTA GAC GG- 3'.

#### Viability assay

Cell viability was measured with the cell proliferation reagent WST-1 (Roche Applied Sciences, Germany). The ready-to-use WST-1 reagent was mixed with cell culture medium (100 μl/mL) and was added to the apical side of the cells for both air-liquid interface and submerged culture conditions. After 30 min incubation at 37°C, the light absorbance at 450 nm was measured.

## Results

### Performance of the nebulizer

The mean volume (or mass) output of the nebulizer was determined by measuring the nebulization time for a known amount of liquid, which was accomplished by observing the clearly visible dense cloud of droplets generated by the nebulizer. For nebulization of 1 mL of salt solution or nanoparticle suspension, the nebulizer needed between 90 and 150 s and the corresponding volume (mass) production rates were between 0.40 and 0.67 mL/min (or 0.40 and 0.67 g/min). The small amount of residual liquid in the nebulizer was disregarded (5-10% for 1 ml of liquid filled into the reservoir volume; measured by gravimetric analysis of the nebulizer before and after discharge). Consequently, the aerosol (droplet) mass concentration was approximately 80-130 g/m^3 ^for a sample flow rate of 5 liter/min.

For consecutive nebulizer runs with 1 mL of 1% NaCl solution and various nanoparticle suspensions, the nebulization times were constant within ± 10 s, which means that the short term repeatability was better than 7%. However, a gradual increase in nebulization time was observed with increasing use of the membrane. For further details on the characteristics of the eFlow nebulizer, please refer to [22,23].

### Aerosol dynamics during ALICE experiments

The droplet deposition on the cells during ALICE exposure was monitored by placing the QCM in the exposure chamber next to the transwell plates containing the cells. As mentioned previously, for liquid films the quantitative interpretation of the change in resonance frequency (*Δf*) of the QCM is altered by various aspects (such as the viscoelastic properties of the film), but the QCM can be used as indicator for mass, if *Δf *remains below 715 Hz, the frequency shift for an (infinitely) thick water layer. By dipping the QCM crystal into water the asymptotic value of 715 Hz was confirmed, as recommended by the manufacturer.

**Figure 3:**
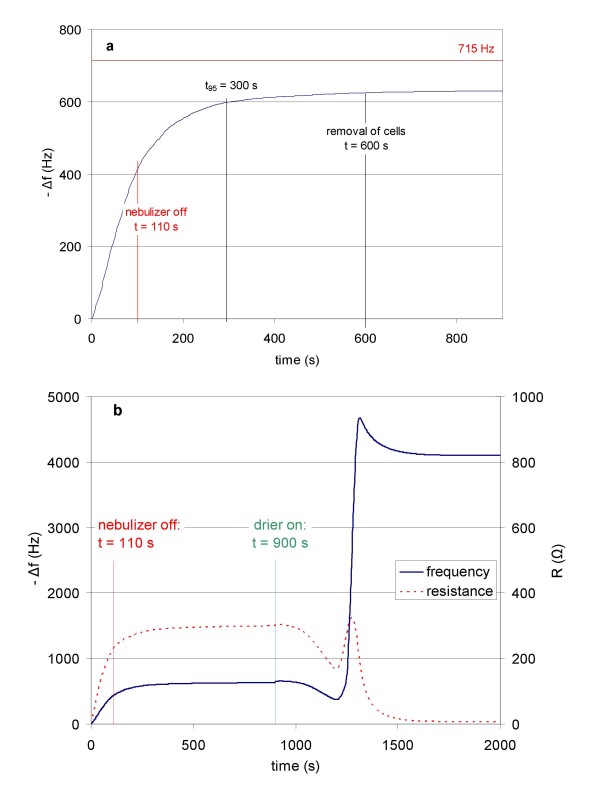
**Response of the quartz crystal microbalance (QCM) during a typical ALICE exposure**. a) Within 2 s of the nebulizer being turned on (t = 0) -*Δf *increases due to droplet deposition on the quartz crystal. At 110 s, the nebulizer is completely discharged (here: 1 mL of 6% (NH_4_)_2_SO_4 _solution) and the air flow is stopped. Subsequently, single particle sedimentation depletes the stagnant cloud and at 300 s, 95% of the final -Δ*f *value (630 Hz) is reached (end of droplet deposition), which is still well below 715 Hz, the saturation value of the QCM for an infinitely thick water layer. During typical ALICE experiments, the cells were removed at 600 s (here no cells were in the ALICE). b) At 900 s dry filtered air is introduced in the exposure chamber, which dries the liquid film on the QCM. At 1500 s, the QCM deposit has completely dried, as indicated by the resistance (*R*) approaching 0 Ω. The dry salt mass of 72.4 μg/cm^2 ^can then be obtained from -Δ*f *= 4100 Hz using equation 3. For optimized timing (~10 min per exposure run) the cells could be removed from the ALICE after 300 s (5 min) and the QCM deposit can be dried more efficiently (within a few minutes) by removing the QCM from the exposure chamber and drying it with dry air.

The response of the QCM during a typical ALICE exposure is depicted in Figure 3. Droplet deposition (increase of -Δf) starts almost immediately (within 2 s) after the nebulizer is turned on (t = 0). At 110 s, the nebulizer is completely discharged (1 mL of 6% (NH_4_)_2_SO_4 _solution was sprayed) and the air flow is stopped. At 300 s, 95% of the final -Δ*f *value (630 Hz) is reached (end of droplet deposition). During a typical ALICE experiment, the cells were removed at 600 s, but data presented in Figure 3 were obtained without cells. No change in -Δ*f *is observed after about 900 s and this value remains constant for hours, if the system is not disturbed (data not shown). At 900 s, the QCM deposit is dried by passing dry filtered air (5 liter/min) into the exposure chamber. The deposit has completely dried at about 1500 s as indicated by the resistance (*R*) approaching 0 Ω, which is a measure for the dissipation of vibrational energy of the quartz crystal due to viscoelastic dampening. At R = 0 Ω, no viscoelastic effects are present, and therefore the deposit is dry. The dry salt mass of 72.4 μg/cm^2 ^can then be obtained from -Δ*f *= 4100 Hz using equation 3. Use of the QCM as real-time indicator for droplet mass deposition is not feasible, if the amount of liquid sprayed exceeds 1 mL, due to the vicinity of -Δ*f *to its asymptotic value (715 Hz).

The data in Figure 3a can be used to identify the lengths of the three phases of the ALICE operation described above (Figure 1). The dense cloud of droplets reaches the bottom of the chamber within 2 s after the nebulizer is activated (end of phase 1). During phase 2 the chamber is gradually filled with droplets until the nebulizer is completely discharged at 110 s (end of phase 2). Phase 3, gradual depletion of the chamber due to single particle settling, is finished after another 190 s.

Hence, the timing of the ALICE experiments can be optimized as follows: The cells and the QCM can be removed from the ALICE after 300 s (5 min) and drying of the QCM deposit outside of the exposure chamber can be accomplished with dry air within a few minutes. Therefore, if the QCM does not need to be cleaned (or if a second clean quartz crystal is available) an entire ALICE run can be performed within ~10 min.

The relevance of cloud settling for the ALICE becomes evident, if the cloud settling and the single particle settling speed are compared. According to Figure 3, the fall time of the cloud to the bottom of the chamber is between 1 and 2 s (onset of -Δ*f *signal), although a more precise determination is not possible due to the sampling frequency of 1 Hz. For a fall distance of 20 cm (distance of inlet port above the ground), this corresponds to 10-20 cm/s. For a droplet concentration of 80-130 g/m^3 ^and a cloud diameter of 1.9 cm (inner diameter of inlet tube), the theoretically expected cloud settling speed is between 13-17 cm/s according to equation 1 (C_D _= 1, [21]), which is in good agreement with the empirical value. Since the single particle settling speed is only 0.077 cm/s (= 4.6 cm/min; 5 μm droplet diameter), the significance of cloud settling for rapid transport of the nebulized substance to the cells is evident. Furthermore, the fact that droplet deposition has ceased almost completely 3 min after the nebulizer has been discharged, suggests that most of the cloud mass resides in the lower half of the exposure chamber, since 5 μm droplets settle about 15 cm in 3 min. This indicates that cloud and single particle settling explain the QCM signal observed in Figure 3a.

### Performance of the QCM for dry deposits

**Figure 4:**
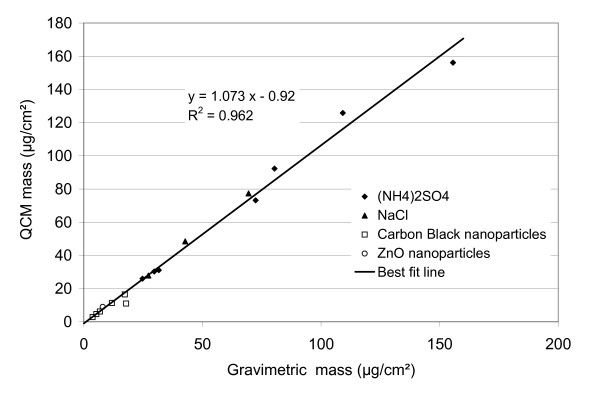
**Comparison of quartz crystal and gravimetric microbalance**. Measurement of the mass deposited on the bottom plate of the ALICE by QCM and gravimetric analysis after nebulization of 1-5 mL of (NH_4_)_2_SO_4_, NaCl, carbon black and ZnO solutions/suspensions with concentrations ranging between 2-10% (salts) and 0.1-2% (carbon black and ZnO nanoparticles). The (dry) mass per surface area as determined by QCM and gravimetry showed excellent linearity (R^2 ^= 0.962) and agreement within 7.3% (slope) over the investigated range from 3 to 160 μg/cm^2^.

As mentioned, the dry nanoparticle/solute mass deposited on the cells can be determined from the dried deposit on the QCM. This was verified by comparing the QCM with gravimetric data for dry (NH_4_)_2_SO_4 _and NaCl, as well as carbon black and ZnO nanoparticles, sprayed in the ALICE, as described above. As seen in Figure 4, both techniques showed excellent linear correlation (R^2 ^= 0.96) and agreement within 7.3% (slope = 1.073). No saturation of the QCM was observed up to 160 μg/cm^2^. This is consistent with the manufacturers provided upper limit of the linear response range of 1770 μg/cm^2^. Measurements below 3 μg/cm^2 ^were impossible due to the detection limit of the gravimetric method.

This confirms the validity of the Sauerbrey equation (equation 3) and shows that the QCM can be used for accurate mass measurements in the ALICE. The validity of the Sauerbrey equation also implies that the prerequisite of the Sauerbrey are met, namely the formation of a uniform, rigid and thin layer on the quartz crystal after exposure in the ALICE.

### Spatial homogeneity and liquid film thickness in the ALICE

**Figure 5:**
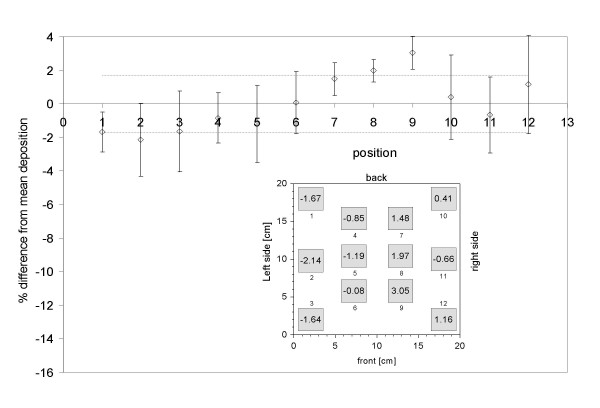
**Spatial uniformity of droplet deposition**. Measurement of (dry) NaCl mass (proportional to droplet mass) deposited on 12 aluminum foils (3 × 3 cm^2^) placed in the exposure chamber (see insert) after nebulization of 1 mL of a 10% NaCl solution in the ALICE. Droplets enter the chamber from the left and the droplet depleted air exits through the right hand side. The data are presented as relative difference from the mean deposited mass on each foil (n = 4) and the variability about the mean. The average of these mean values is 0% (per definition) and the variability of the mean values (spatial uniformity) is 1.6% (dashed lines). Since this is identical to the estimated measurement uncertainty, the data indicate uniform particle deposition across the exposure chamber.

The good agreement between the QCM and the gravimetric mass already suggests that the droplet deposition is spatially homogeneous on the sensitive part of the quartz crystal (0.4 cm^2^), since this is a pre-requisite for the validity of the Sauerbrey equation. This important issue was investigated more rigorously by distributing 12 rectangular pieces of aluminum foils (3 × 3 cm^2^) over the exposure chamber (see insert in Figure 5), while spraying 1 mL of 10% NaCl solution into the exposure chamber following the standard ALICE procedure described above. On average, 1.45 mg NaCl was deposited per foil, which corresponds to 162 μg/cm^2^, and the gravimetric analysis of the dried foils (n = 4) revealed that the observed spatial variability was 1.6%. Since this value is consistent with the estimated measurement accuracy (1.6%), no statistically significant spatial uniformity was found in the exposure chamber.

**Figure 6:**
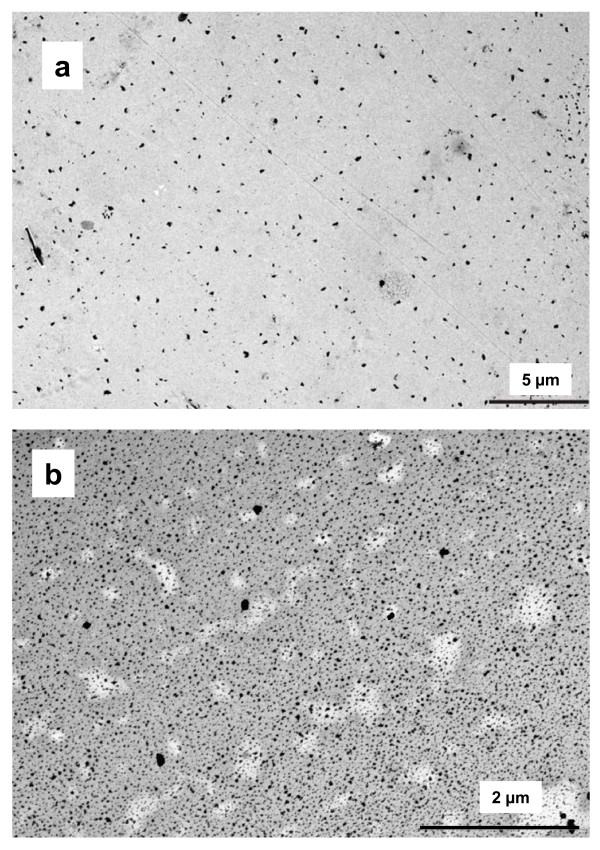
**TEM micrograph of ZnO nanoparticles deposited in the ALICE**. Depicted are representative micrographs obtained after nebulization of 1 mL of ZnO suspension (primary diameter 24-71 nm) with two different concentrations, a) 0.3 mg/mL and b) 7.5 mg/mL. As expected, the ZnO coverage increases with ZnO concentration and, although larger agglomerates are starting to appear for the higher concentration, their fractional contribution is still small. In both cases the deposition pattern is uniform not only on the mm scale as shown in Figure 5, but also on the scale of single droplets (micron scale). This is consistent with the formation of a continuous 14 μm liquid film after nebulization of 1 mL of suspension (estimated from deposition efficiency and chamber geometry), which implies that more than 4 droplets deposit on each location of the chamber.

The high degree of homogeneity was also confirmed for Au and ZnO nanoparticles by placing a TEM grid in the ALICE. Since similar results were obtained for both nanoparticle types, only the ZnO data are shown here. As seen from Figure 6, the ZnO coverage increases with the ZnO concentration in the suspension and, although larger agglomerates are starting to appear for the higher concentration, their fractional contribution is still small. This indicates that no substantial particle agglomeration has occurred during the ALICE experiment, even for the highest ZnO concentration. In contrast, ZnO agglomeration is not negligible during submerged exposure, since agglomeration is enhanced in the presence of cell culture medium [29] as seen by the formation of large agglomerates (900 nm) within 30 min as determined from dynamic light scattering measurements described above.

It is also evident from Figure 6 that the deposition pattern is uniform for both concentrations and no micron-sized patches or "hot spots" of nanoparticles are present as might be expected after deposition of individual micron-sized droplets (MMD = 4.4-5.4 μm). This can be rationalized by considering the thickness of the liquid layer on the grids (cells) after ALICE exposure. From the deposition efficiency evaluation of the droplets in the chamber (0.57 ± 0.07 as determined below) and the area of the ground plate of the exposure chamber (400 cm^2^), we find that a continuous 14 μm liquid layer is formed in the ALICE for 1 mL of nebulized suspension. For 5 μm (MMD) droplets, this means that on average 4.2 droplets are falling on each location of the exposure chamber. The theoretical minimum thickness of a continuous layer (perfectly uniform deposition of drops with 1 drop per location) is 3.3 μm (=2/3 MMD), which corresponds to 0.24 mL of sprayed liquid. However, if a continuous layer is desired, spraying at least 0.5 mL is recommended in order to compensate for small variations in the deposition pattern.

### Efficiency and repeatability of droplet deposition

**Figure 7:**
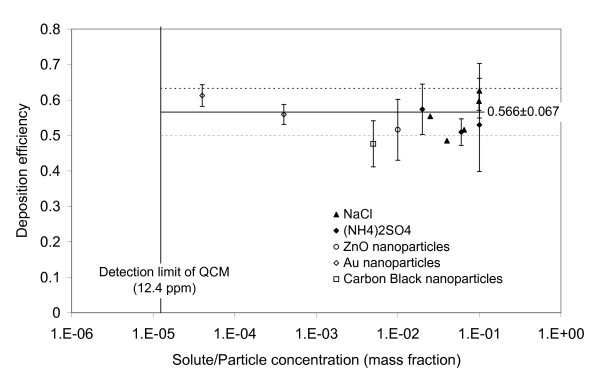
**Deposition efficiency of nanoparticles and solutes on the bottom of the exposure chamber**. All measurements were performed after nebulization of 1 mL of suspension/solution with varying nanoparticle/solute mass concentration. The symbols represent the mean and standard deviation (error bars) for up to 12 independent measurements (no error bar for n = 1). The solid and dashed lines indicate the mean (0.566) and standard deviation (0.067) of the entire data set, respectively. The 68% confidence level (SEM) of the mean is 0.012 (n = 30). The detection limit of the QCM (12.4 ppm) corresponds to 18 ng/cm^2^.

Another important characteristic of the ALICE is the deposition efficiency of the nebulized material on the bottom of the exposure chamber or even more importantly on the cells. Since the deposition efficiency on the cells depends on cell coverage and hence on the type of transwell plates used, the deposition efficiency was initially investigated in the exposure chamber, which is defined as the ratio of solute (or nanoparticles) mass deposited on the bottom plate and solute/nanoparticle mass filled into the nebulizer. As seen from Figure 7, the mean and standard deviation of the deposition efficiency was 0.57 ± 0.07, independent of the type of solution (NaCl, (NH_4_)_2_SO_4_) or nanoparticle suspension (ZnO, Au). Gravimetric analysis indicated that 5-10% and 15-20% of the liquid remained in the nebulizer and the exit filter, respectively. The unaccounted remainder of 10-20% must have been deposited in the connecting tubing and the side/top walls of the exposure chamber. Since the cell-specific deposition efficiency depends on the fractional cell coverage of the exposure chamber (400 cm^2^), the deposition efficiency on the cells is lower than 0.57. If two standard plates with 6-, 12- or 24-transwell inserts are placed in the ALICE, 50.4, 21.6 and 14.4 *cm*^2 ^of the exposure chamber are covered with cells resulting in a cell-specific deposition efficiency of 0.072, 0.031 and 0.021, respectively.

The standard deviation (0.07) of the deposition efficiencies (n = 30) represents the repeatability of substance delivery to the cells in the ALICE (Figure 7). Since the mean deposition efficiency is 0.57, the repeatability of the ALICE is 12% (=0.07/0.57) for the solutions and suspensions investigated here.

It is noteworthy that the deposition efficiency is independent of the amount of sprayed material as was confirmed for 1 mL to 5 mL salt solutions and nanoparticle suspensions. This indicates that all "loss mechanisms" are independent of time including the depletion of the sample flow due to cloud settling.

### Cell exposure experiments

#### Effect of ALICE exposure on cell viability

**Figure 8:**
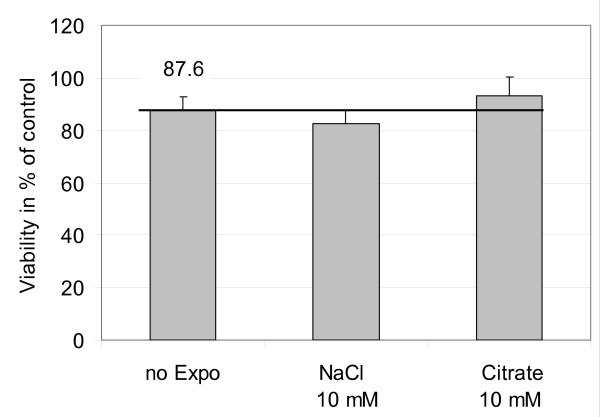
**Effect of cell handling on cell viability during ALICE experiments**. Cell viability (WST-1 test; 3 h incubation after exposure) was measured in immortalized human alveolar epithelial-like cells (A549). "No expo" cells were treated identical to ALICE cells except that they were not put into the ALICE and instead they remained in the incubator. Cells maintained under submerged conditions served as a control (100%). Cell viability was slightly impaired for all cases (87 ± 2%), but there was no statistically significant difference between 10 mM NaCl- and citrate-exposed cells and not exposed cells. Hence, the slightly reduced cell viability (relative to submerged conditions) is likely due to culturing the cells for about 18 h at the air-liquid interface prior to exposure, but not due to cell handing in the ALICE.

Potential adverse effects of cell handling on cell viability (WST-1 test) during ALICE experiments were investigated with A549 lung epithelial cells. Although the viability of the cells was slightly impaired (87 ± 2% relative to submerged cells; Figure 8) after exposure to 1 mL of 10 mM aqueous NaCl (0.9 μg/cm^2 ^NaCl) and 10 mM tri-sodium citrate dihydrate solution (4.4 μg/cm^2 ^citrate), there was no significant difference between exposed and non-exposed cells. The somewhat reduced viability was likely due to the 18 h adaptation of the cells to the air-liquid interface conditions (prior to exposure) and not due to the ALICE exposure procedure. It is noteworthy that slight reductions of cell viability after transfer of submerged cells to the air-liquid interface were also reported by other investigators ([19,30,31]). Consequently, neither cell handling in the ALICE nor exposure of the cells to salt solutions impaired cell viability.

#### Comparison of cellular response under submerged and air-liquid interface conditions after ZnO exposure

As an application of the ALICE system, A549 cells were exposed to various doses of ZnO nanoparticles and the mRNA expression of the pro-inflammatory cytokine IL-8 and the oxidative stress marker HO-1 were investigated. Cell viability (WST-1) was not impaired for any of the particle concentrations used here (data not shown). The ratio of mRNA levels from the air-liquid interface and submerged conditions in the absence of ZnO was 1.9 and 1.4 for IL-8 and HO-1, respectively. This reflects the effect of transferring the cells to the air-liquid interface conditions. Furthermore, since there was no significant difference between non-exposed and 10 mM citrate-exposed cells at the air-liquid interface (data not shown), both non-exposed and 10 mM citrate-exposed cells can be used as negative control for ALICE experiments.

**Figure 9:**
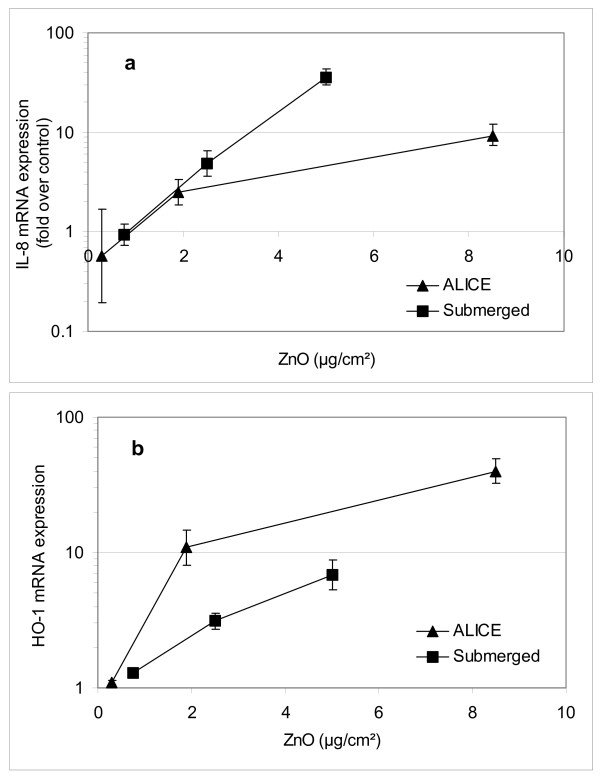
**Cellular dose-response due to ZnO nanoparticle exposure under submerged and air-liquid interface conditions**. The dose-dependent IL-8 (pro-inflammatory) and HO-1 (oxidative stress) mRNA expression in A549 cells was measured with qRT-PCR after ZnO exposure in the ALICE (3 h post-exposure incubation period; control: 10 mM citrate) and under submerged conditions (for 3 h; control: pure medium). Reported are the geometric mean and standard deviation (n>3). None of the responses was statistically significant below 1.0 μg/cm^2^. For larger doses, significant differences between ALICE and submerged responses were observed.

ALICE experiments were performed with ZnO nanoparticle doses of 0.3, 1.9 and 8.5 ZnO μg/cm^2 ^(n ≥ 3). For reference, ZnO exposures under submerged conditions were also conducted at 0.7, 2.5 and 5.0 ZnO μg/cm^2 ^(n ≥ 4). As discussed above, the ZnO dose was determined from the amount of ZnO nanoparticles added to the culture medium. As seen in Figure 9, the IL-8 and HO-1 mRNA expression increased with ZnO dose for both ALICE and submerged conditions, but none of the responses was statistically significant below 1.0 μg/cm^2^. For IL-8, the ALICE and the submerged data agreed within experimental uncertainties except for the highest dose, where a higher response was observed under submerged conditions (Figure 9a). In contrast, HO-1 was clearly more induced in the ALICE except for the lowest concentration. Hence, the cellular dose-response for IL-8 and HO-1 was different under air-liquid interface and submerged conditions.

## Discussion

Due to the well-known shortcomings of submerged cell exposure systems for pharmaco-toxicological studies on pulmonary epithelial cells (such as uncertain effective biological dose for nanoparticles and unrealistic exposure scenario due to missing air-liquid interface and absence of mucus or lining fluid on respiratory epithelial cells), there have been several approaches to develop exposure systems for cells at the air-liquid interface. In the CULTEX system, particles are deposited from a continuous aerosol flow by diffusional and gravitational deposition onto cells [14]. Bitterle and coworkers refined this approach by establishing a stagnation point flow over the cell layer, which enhanced the deposition efficiency of 200 nm submicron sized particles from about 0.7% [32] to 2% [13]. Deposition efficiencies of 15-30% were obtained for charged 50-600 nm particles under the influence of an alternating electrostatic field [16] or in the EPDExS system utilizing unipolar electrostatic deposition downstream of a differential mobility particle sizer [33]. None of these types of devices was designed for nebulized liquid substances (micron-sized droplets) and none of them (except for one Bitterle type system using a QCM for dose measurement [34]) allows for real-time, direct measurement of the amount of particles deposited onto the cells, but rather infer the cell deposited dose indirectly from the product of the measured particle concentration in the air and the empirically determined deposition efficiency.

Previously described methods for deposition of micron-sized particles or droplets onto cells have relied on inertial impaction as the deposition mechanism. The use of impactors or impingers [15,17,18,35] requires high flow rates for effective particle deposition, which may impair cell viability [36] and the deposition efficiency is also strongly dependent on particle size [21]. While the high flow speed (30 cm/s in the device by [17]) may simulate *in vivo *conditions in the respiratory bronchioles after a sharp inspiration [17], it exceeds typical flow speeds under normal breathing conditions and is completely unrealistic for the alveolar regime, where flow velocities are so small that deposition due to impaction can be disregarded [37]. A second type of cell exposure system for liquids utilizes high velocity sprays to deposit the liquid directly onto the cells via inertial impaction [19,20]. High-speed spray-deposition does not occur under *in vivo *conditions (except in the mouth after using a medical spray nebulizer), and both systems may induce cellular stress due to high speed droplet collisions. Again, none of the systems above provides information on the cell deposited dose.

The ALICE utilizes a completely different, more "gentle" technique of particle generation and deposition and it provides direct dose measurements. As discussed above, micron-sized droplets (MMD = 4.5-5.4 μm) are generated at a very high concentration (80-130 g/m^3^) by a vibrating membrane nebulizer and are uniformly deposited via cloud and single particle settling onto cells at the air-liquid interface. It is noteworthy that the cloud settling speed of 13-17 cm/s is too small for impaction to occur, as evidenced by the absence of enhanced droplet deposition near the center of the chamber, where the cloud speed is largest (see foils 5 and 8 in Figure 5). While impaction is a relevant deposition process in the upper respiratory tract, gravimetric sedimentation simulates the *in vivo *conditions of supermicron particle deposition in the alveolar region. The gentle form of particle deposition (compared to impaction) minimizes mechanical strain and subsequent stress for the exposed biological material, as was confirmed by the unaffected viability of human alveolar epithelial A549 cells after ALICE exposure (Figure 8). Furthermore, none of the ZnO nanoparticle doses applied here had any significant effect on cell viability, which is a pre-requisite for reliable measurements of particle-induced oxidative stress response, as performed in the current study.

In contrast to jet nebulizers or ultrasonic generators, vibrating membrane nebulizers do not exert high shear forces and do not heat the nebulized liquid during the generation process. Hence, it does not jeopardize the biological activity of potentially delicate therapeutic agents, such as biopharmaceuticals [23,38]. Other positive aspects of the eFlow technology are the low residual volume in the reservoir (5%-10%), the large and stable mass/volume output (80-130 mg/m^3 ^for 5 liter/min flow rate) and the low electrostatic charging of the droplets. Furthermore, the near micron-sized membrane pores may prohibit supermicron particles and nanoparticle agglomerates to exit the nebulizer. This could explain the measured reduced deposition efficiencies (0.3-0.4) for ZnO nanoparticle suspensions, which were not freshly prepared prior to nebulization. The prolonged residence time of the ZnO nanoparticles in suspension allowed for enhanced agglomeration (as confirmed by DLS measurements) leading to supermicron-sized agglomerates, which were too large to pass through the pores of the nebulizer membrane. Due to the increasing interest in the pharmacological and toxicological effects of nanoparticles, the prevention of large agglomerates of nanoparticles from getting delivered to the cells may be an attractive feature of the ALICE, which comes at the expense of reduced deposition efficiency (less than 0.57 ± 0.07).

The QCM is a highly sensitive, fast-response instrument for real-time determination of the deposited substance mass on the cells. The QCM has a response time of ~1 s, a large dynamic range (0.018-1800 μ*g*/*cm*^2^; extendable to larger mass with non-linear correction factors) and high accuracy (here: <7.3% agreement with gravimetry). Its drawback for droplet measurements is that the QCM is sensitive to viscoelastic effects, which requires drying of the deposited droplets for accurate determination of the deposited nanoparticle/solute mass. However, the current study has shown that the QCM can be used as a real-time indicator for droplet deposition as long as the deposited liquid layer induces a frequency shift (QCM signal) of less than 715 Hz, the asymptotic value of an ''infinitely'' thick water layer. Finally, it must be stipulated that for stabilized nanoparticle suspensions, the (dry) nanoparticle mass can only be obtained from the QCM data, if the mass ratio of nanoparticles and stabilizing agent is known.

The repeatability of 12% in mass dose delivery is similar to the value of about 10% reported for the spray exposure unit RHINOCON ([19]; error bar of the mean deposition in their figure 2). The entire area of the ALICE chamber can be used for cell exposure experiments, since the spatial variability of the ALICE is small (better than 1.6%). For comparison, the spatial variability of the RHINOCON system was 8% [19], which is probably due to the less uniform spray produced by commercially available spray units. In spite of excellent uniformity in the ALICE, agglomeration of nanoparticles, both prior and after nebulization of unstable nanoparticle suspensions, cannot be ruled out. Hence, for unstable nanoparticle suspensions the authors recommend minimization of potential agglomeration by reducing the processing time (less time for agglomeration), choosing low nanoparticle concentrations (less collision probability) and providing visual confirmation of spatial uniformity following nebulization (TEM measurements).

Although the deposition efficiency in the exposure chamber of the ALICE is relatively large (0.57 ± 0.07), the cell-specific deposition efficiency is currently limited to 0.072 (for two 6-well plates) due to the poor fractional cell coverage of standard transwell plates. For comparison with previously described cell exposure systems, it is important to note that the deposition efficiency of 0.072 represents the *overall *deposition efficiency, which is defined as the ratio of cell deposited and total mass of the substance filled into the nebulizer. Previous studies typically reported the *internal *deposition efficiency, the fractional deposition of the substance entering the exposure system, which does not account for reduced overall deposition efficiency due to, for example, residual substance in the nebulizer (particle generator) or substance loss in the conductive tubing upstream of the exposure chamber. The overall deposition efficiency is the more relevant parameter for materials, which are expensive or in limited supply (e.g. modern drugs), since it provides the basis for estimating the true costs of exposure experiments.

Unfortunately, previous exposure systems have either not been characterized in terms of deposition efficiency (no deposition efficiencies are given for any of the cell exposure systems for liquid substances described in the literature [18-20]) or the reported deposition efficiencies refer to the *internal *deposition efficiency, that is it discards residual material in the particle generator or losses upstream of the exposure system. For purely diffusion-based cell exposure systems, the internal deposition efficiencies were limited to 0.02 (relative to the dose entering the exposure system). Internal deposition efficiencies near unity (100%) were reported for electrostatic deposition of charged particles. Stevens and coworkers [33] deposited size-selected charged particles by passing the aerosol through a bipolar charger and subsequently through a differential mobility analyzer (DMA). Since the charging efficiency of a bipolar charger is limited to about 0.30 [21], the overall deposition efficiency is limited to about 0.3. For the bipolar electrostatic deposition system by Savi and coworkers [16], deposition efficiencies of 0.15-0.30 were reported. However, all of these values are upper limits for the overall deposition efficiency, since they do not account for additional losses due to, for example, residual mass in the particle generator (which can be large depending on the particle generator), losses in the transport lines and waste material during turn-on/turn-off phase of the exposure system. Hence, the overall deposition efficiency of 0.072 for the ALICE is better than, or within the range of, typically reported upper limits of overall deposition efficiencies for air-liquid cell exposure systems.

After nebulization of 1 mL of liquid, a 14 μm liquid layer is formed on the cells in the ALICE. Since the layer thickness is small compared to the diameter of the transwell inserts for the cells (>6.4 mm for standard 6-, 12-, and 24-well plates), nearly 100% of the nanoparticles (or solute molecules) interact with the cells due to diffusional motion and diffusional losses to lateral walls are small. Furthermore, *in vivo *epithelial cells are typically covered by a thin liquid layer. Hence, generation of a thin liquid film on the ALICE cells prevents evaporation of liquid from the cells and resembles physiological conditions, especially since the thickness of the deposited film can be regulated by epithelial cells via water transport to the basal side [39].

Although the nebulizer used in the current study can spray up to 5 mL of liquid per filling, the authors recommend using 1 mL for the following reasons: The QCM can be used as real-time indicator for 1 mL, or less, of sprayed liquid (frequency shift <715 Hz), the deposited liquid layer is sufficiently thin (14 μm) for efficient nanoparticle-cell interaction and the corresponding exposure time is short (5 min, which increases by about 2 min per additionally sprayed mL of liquid). Using less liquid reduces the liquid layer thickness and exposure time, but it also enhances the residual liquid fraction remaining in the nebulizer (0.05-0.1 mL; 10-20% for 0.5 mL) and hence the cell deposition efficiency.

The suitable concentration range of the nanoparticle suspension used in the ALICE is determined by the detection limit of the QCM. For nebulization of 1 mL of liquid, the lower detection limit of the QCM (0.018 μ*g*/*cm*^2^) corresponds to a solute/nanoparticle concentration of 12.4 ppm (mass) in water. Assuming a maximum solute/nanoparticle concentration of 10%, a dose of 160 μg/cm^2 ^per ALICE run can be supplied to the cells. The lowest concentration applied in the ALICE as yet, was 40 ppm of 15 nm gold nanoparticles, which resulted in a dose of 0.061 μg/cm^2 ^(Figure 7). This dose level and a 10-fold higher dose were used by Brandenberger and coworkers who applied the ALICE to study cellular uptake and toxicological effects of a triple cell co-culture model simulating the alveolar lung epithelium due to gold nanoparticle exposure at the air-liquid interface [40].

Although air-liquid interface exposures have become more widely used in recent years, there are very few quantitative comparisons between air-liquid interface and submerged (conventional) dose-response curves after nanoparticle exposure. Since the ALICE provides direct accurate dose measurements, the data set provided here can be used for such a comparison. For both exposure conditions, A549 cells showed no significant response in IL-8 and HO-1 mRNA expression after exposure to less than 1.0 μg/cm^2 ^ZnO nanoparticles (Figure 9). In contrast, significant differences between exposure methods were observed for larger concentrations. For the highest investigated dose (submerged: 5.0 μg/cm^2^; ALICE: 8.5 μg/cm^2^), the ratio of ALICE and submerged response was 0.26 and 5.7 for IL-8 and HO-1, respectively. This indicates a substantially mitigated and enhanced response for the ALICE relative to submerged conditions for the pro-inflammatory (IL-8) and oxidative stress marker (HO-1), respectively. The underlying reasons for these differences are currently unknown.

To put the dose levels obtained during *in vitro *exposures (ALICE: 0.3 - 8.5 μg/cm^2^; submerged: 0.75 - 5 μg/cm^2^) into perspective, it is instructive to consider that the current recommended Occupational Safety and Health Administration (OSHA) standard for ZnO fumes is 5 mg of ZnO per cubic meter of air (5 mg/m^3^) averaged over an eight hour work shift. Assuming an accumulated breathing volume of 3 m^3 ^in 8 h, a lung surface area of 140 m^2^, an alveolar deposition efficiency of 10-50% (depending on particle size) and a 70% long-term clearance from the alveolar regime [41] the OSHA standard corresponds to an average (long-term) daily alveolar surface dose of 0.32-1.6 ng/cm^2^. Hence, the maximum lifetime dose accumulated by a worker is 3.6-18 μg/cm^2 ^(5 workdays per week for 50 weeks per year over 45 years). The upper limits used for the current *in vitro *experiments are within this lifetime range and the lowest submerged dose of 0.75 μg/cm^2 ^still corresponds to several years of exposure at the maximum allowed dose level. Since no significant *in vitro *cellular response was observed after challenging the A549 cells with 0.75 μg/cm^2 ^(for 3 h post-incubation time), the current data suggest that ZnO nanoparticles do not pose a significant health risk, if the OSHA exposure limits are obeyed. However, it is unclear whether this result also holds for a chronic exposure scenario, that is continuous delivery of the mean daily dose (0.32-1.6 ng/cm^2^) over a lifetime.

## Conclusions

The air-liquid interface cell exposure system (ALICE) allows uniform, efficient and dose-controlled deposition of solutions and nanoparticles in liquids on cell systems. The ALICE is a closed system suitable for up to two commercially available cell culture plates, which can be operated by a technical assistant without expert knowledge in aerosol technology. The deposition mechanism of the ALICE is based on gravimetric sedimentation, which simulates the *in vivo *conditions in the alveolar region. The pore size of the nebulizer membrane limits the size of the cell deposited nanoparticles (agglomerates) to about 1 μ m. Although the ALICE was used here for a cell line, it is also applicable to other types of biological material such as tissue sections or even microorganisms.

Cell exposure in the ALICE had no adverse effect on the viability of a widely used alveolar epithelial cell line (A549) for the recommended amount of liquid to be sprayed (1 mL). Dose delivery was repeatable (12%), uniform (<1.6% spatial variability) and occurred at a total (net) cell-specific deposition efficiency of up to 0.072 (for two 6-well plates). While this may seem small, it exceeds the total deposition efficiency obtained with most other exposure systems, if one includes all possible losses in the balance including residual liquid in the nebulizer, transport losses and losses during the turn-on/turn-off phase. Currently, the deposition efficiency of the ALICE is mainly limited by the low fractional cell coverage of standard transwell plates. For ideal cell coverage (100%) in the exposure chamber, the deposition efficiency can be improved to 0.57.

Dose-measurements with a quartz crystal microbalance (QCM) allow fast (~10 min for exposure and mass determination) and accurate (<7.3%) determination of the cell deposited dose. For the recommended 1 mL of sprayed liquid, a single ALICE exposure can provide 0.02-200 μg/cm^2 ^of substance to the cells depending on the mass concentration of the nanoparticles/solute (0.0012-12%).

The applicability of the ALICE to obtain dose-response curves was demonstrated by exposing A549 cells to various doses of ZnO nanoparticles. The mRNA expression of the pro-inflammatory cytokine IL-8 and the oxidative stress marker HO-1 indicated substantial differences depending on the exposure method (submerged versus air-liquid interface) for doses larger than 1 μg/cm^2^. However, both methods suggest that ZnO nanoparticles are not toxic, if occupationally allowed exposure levels are obeyed.

The small amount of liquid to be used (1 mL), the gentle method of droplet generation, the realistic deposition process (for the alveolar region) as well as the competitive deposition efficiency (0.072) make the ALICE a useful *in vitro *tool for pharmacological and toxicological studies for a wide variety of substances including newly developed expensive and/or delicate drug formulations for e.g. gene transfer therapy.

## Competing interests

The authors declare that they have no competing interests.

## Authors' contributions

GAF, BL, HS, EK and OS designed the exposure chamber, AGL, GAF, OS, EK conceived and designed the experiments; AGL, GAF, BL, VD, and CB performed experiments; AGL, EK and OS performed data analysis; AGL, CB, BRR, and OS made substantial contributions to writing the manuscript. All authors read and approved the final manuscript.
